# An evaluation of Internet searches as a marker of trends in population mental health in the US

**DOI:** 10.1038/s41598-022-12952-5

**Published:** 2022-05-27

**Authors:** Uma Vaidyanathan, Yuantong Sun, Tomer Shekel, Katherine Chou, Sandro Galea, Evgeniy Gabrilovich, Gregory A. Wellenius

**Affiliations:** 1 Independent Researcher, Washington, DC, United States; 2 Department of Environmental Health, Boston University School of Public Health, Boston, MA USA; 3 Google Health, Palo Alto, CA USA; 4 Boston University School of Public Health, Boston, MA USA

**Keywords:** Human behaviour, Public health

## Abstract

The absence of continuous, real-time mental health assessment has made it challenging to quantify the impacts of the COVID-19 pandemic on population mental health. We examined publicly available, anonymized, aggregated data on weekly trends in Google searches related to anxiety, depression, and suicidal ideation from 2018 to 2020 in the US. We correlated these trends with (1) emergency department (ED) visits for mental health problems and suicide attempts, and (2) surveys of self-reported symptoms of anxiety, depression, and mental health care use. Search queries related to anxiety, depression, and suicidal ideation decreased sharply around March 2020, returning to pre-pandemic levels by summer 2020. Searches related to depression were correlated with the proportion of individuals reporting receiving therapy (r = 0.73), taking medication (r = 0.62) and having unmet mental healthcare needs (r = 0.57) on US Census Household Pulse Survey and modestly correlated with rates of ED visits for mental health conditions. Results were similar when considering instead searches for anxiety. Searches for suicidal ideation did not correlate with external variables. These results suggest aggregated data on Internet searches can provide timely and continuous insights into population mental health and complement other existing tools in this domain.

## Introduction

Syndromic surveillance systems aim to provide near real-time assessment of influenza-like illness, gastrointestinal or respiratory symptoms, or other specific signs or symptoms of physical health^1–4^. The COVID-19 pandemic further highlighted the value of real-time insights into population health and wellbeing. For example, models forecasting COVID-19 cases, hospitalizations, and deaths substantially aided the public health response to the pandemic^5^. While a variety of existing surveys^6–11^ have provided insights into population mental health in a specific place or moment, none offer near-real time insights into population mental health across a wide geographic area prior to and during the pandemic time frame.

Though initial cases of COVID-19 were documented in China in late 2019, the threat of a global pandemic rose to the world’s public consciousness in early 2020. In the US, stay-at-home orders and other mitigation measures of varying strictness were enacted across much of the country starting in March 2020. The impacts of these restrictions on population mobility and time spent at home have been previously documented^12^.

There has been substantial interest in assessing the impacts on population mental health of the COVID-19 pandemic and the public health measures enacted to control the spread of the novel coronavirus. For example, some studies suggest that the pandemic led to more people experiencing symptoms of anxiety and depression^13–15^, and to a surge in telehealth visits for mental health^16^. Other studies show less clear results, with reports of moderate impact, no impact, or even an improvement in particular markers of mental health or in subsets of the population^17–22^. Internet search patterns may provide valuable insights into population mental health in near-real time and at unprecedented scale. However, this possibility has not been comprehensively evaluated and results of prior studies have yielded mixed results^23–27^. Indeed, both the opportunities and challenges of using Internet search data in this way are widely recognized^28,29^.

In this study, we sought to evaluate the utility of Google’s COVID-19 Search Trends Symptoms Dataset^30,31^ (hereafter referred to as the Symptom Search Dataset or SSD) to gain insights into population mental health. SSD is distinct from the well-known Google Trends (https://trends.google.com/) product in that SSD is specifically focused on quantifying the volume of searches related to medical and mental health symptoms and conditions rather than specific manually identified queries, and is available at the finer spatial granularity of US counties. We compared data on Internet searches related to mental health across the US to other available instruments for assessing population mental health, and subsequently used search patterns to gain insights on the impact of the COVID-19 pandemic on population mental health in the US.

## Methods

We utilized publicly available, anonymized, and aggregated national-level data from Google’s Symptom Search Dataset (SSD), which reports on the relative frequency of Internet searches for 420 signs, symptoms, and health conditions with well-documented privacy protections^31^. For comparison, we used data from: (1) the Centers for Disease Control and Prevention’s (CDC) National Syndromic Surveillance Program (NSSP), which tracks emergency department (ED) visits for various health conditions from facilities across 48 US states^6^ and (2) the US Census Bureau’s Household Pulse Survey (HPS) assessing the social and economic impact of the pandemic^7^. The key features of these data sets are summarized in Table 1.

**Table 1:** Summary of features of the Google COVID-19 Search Trends symptoms, National Surveillance Syndromic Program, and Household Pulse Survey datasets used in the current study.

Dataset	Variables	Time range	Sample demographic	Sample size
Google COVID-19 Symptom Search Dataset	Searches related to: (1) Anxiety (2) Depression (3) Suicidal ideation (4) Motion sickness	01/01/2018–04/04/2021 (171 weeks)	US-wide Google search engine users	Tens of millions of users
US Census Household Pulse Survey	Symptoms of (1) Anxiety (2) Depression	04/27/2020–3/29/2021 (27 weeks)	US-wide nationally representative Internet survey	Sample size: Range: 39,447–118,897
				Weighted response rate: Range: 1.3–10.3%
	For mental health concerns, proportion reporting: (1) Taking prescription medication, (2) Receiving counseling or therapy, or (3) Unmet need	08/19/2020–3/29/2021 (15 weeks)	US-wide nationally representative Internet survey	
National Syndromic Surveillance Program	Count of emergency department (ED) visits for age 10+ for: (1) Disaster related mental health, and (2) Suicide attempts	12/30/2018–10/04/2020 (93 weeks)	Data from ED facilities in 48 US states and Washington DC	(1) Average # of ED visits for disaster related mental health: 40,192 (range: 30,034–45,618)
				(2) Average # of ED visits for suicide attempts: 4836 (range: 4064–5606)

### Data sources

SSD is publicly available^30^ and provides daily and weekly time-series of the relative volume of searches in the United States in English or Spanish for common symptoms and conditions. The data are available at national, state, and county levels in the US and five other English-speaking countries. Search queries relating to each symptom are aggregated and anonymized through the use of differential privacy^32^, and then normalized by the total search volume in that region, as detailed elsewhere^31^.

SSD was created by leveraging Google’s web search tools that map queries onto Knowledge Graph^33,34^ entities by continuously learning the associations between words in user queries and the entities described in web pages viewed following those queries. The 420 symptoms and conditions included in SSD represent the most frequently searched entities (by query volume). Each entity (symptom or condition) is associated with tens or hundreds of thousands of individual queries issued by Google users on desktop computers or mobile devices. Quotation marks and capitalization in queries are ignored and spelling mistakes are autocorrected. Sample queries included [lexapro], [depression test], or [signs of depression] for depression; [trazodone], [agoraphobia] or [panic attack] for anxiety; and [I want to die], [how to die] and [I want to kill myself] for suicidal ideation.

For the current study, we focused on SSD search queries related to anxiety, depression, and suicidal ideation between January 1, 2018 through December 31, 2020. We chose these entities a priori because they represent common conditions that are frequently searched for, and because of their high relevance to population mental health. We also considered searches related to motion sickness as a putative negative control in a subset of our analyses.

We compared national-level, weekly data on Internet searches as measured by SSD to national-level data on ED visits as reported by the NSSP. The NSSP is a collaboration led by the CDC to collect, analyze, and share electronic health data from approximately 3500 emergency departments, urgent and ambulatory care centers, inpatient healthcare settings, and laboratories (collectively referred to as ED facilities from here on) across 48 states (excluding Hawaii and Wyoming) and Washington DC^6^. These facilities account for approximately 70% of all US ED facilities. The data used in this analysis were previously utilized by Holland et al. (2021)^20^ and reused in the current study with permission from the authors.

We focused on two variables reported on by Holland et al. (2021)^20^: (1) national counts of weekly ED visits for mental health conditions associated with natural or human-originated disasters, such as stress, anxiety, symptoms consistent with acute stress disorder or posttraumatic stress disorder, and panic, and (2) national counts of weekly suicide attempts. The dataset included weekly ED visit counts from December 30, 2018 to October 10, 2020.

We additionally compared Internet search data to data from the HPS. The HPS is a national survey designed to measure the impacts of the COVID-19 pandemic on the economic, physical, and mental health of American households^7^. Phase 1 of the survey took place between April 23, 2020 and July 21, 2020, Phase 2 took place from August 19, 2020 to October 26, 2020, and Phase 3 took place between October 28, 2020 and March 29, 2021. Although the survey is still ongoing, in the current analysis we used HPS data from these three phases^35^.

Questions regarding symptoms of anxiety and depression were administered in all phases of the survey, while questions regarding mental health care were included in Phases 2 and 3. Questions regarding symptoms of anxiety and depression included 4 items that are a modified version of the two-item Patient Health Questionnaire (PHQ-2) and the two-item Generalized Anxiety Disorder (GAD-2) questionnaires. For each question, responses covered the last 7 days and were coded as follows: not at all = 0, several days = 1, more than half the days = 2, and nearly every day = 3. Scores for anxiety and depression were obtained by summing responses across the two questions for each construct. The percentage of respondents scoring 3 or above on these summed scores is used in analyses of survey results. Items indexing mental health care assessed the percentage of adults in the past 4 weeks that reported taking prescription medication, receiving counseling or therapy from a mental health professional, or needing counseling or therapy from a mental health professional but not receiving it (i.e., unmet needs).

### Statistical analyses

We first used graphical approaches and descriptive statistics to identify temporal patterns in Internet searches related to anxiety, depression, and suicidal ideation. We then fit a generalized linear model with a log link function to quantify the impacts on relative search volumes associated with the week of the Thanksgiving and Christmas holidays and the onset of the COVID-19 pandemic (defined as the first 4 weeks of March 2020), adjusting for calendar year and season.

Second, we quantified the change in search volumes associated with the pandemic by calculating the percent change in search frequency for each topic versus the same week 1 year earlier for the period from January 1, 2020 through December 31, 2020. We similarly estimated the change in rates of ED visits for mental health symptoms and suicide attempts from the NSSP.

Third, we computed pairwise Pearson correlation coefficients between contemporaneous measures derived from SSD, NSSP, and HPS. Results were not materially different when using Spearman rather than Pearson correlation coefficients. We additionally used scatter plots to visualize the relationship between specific pairs of markers in more depth. In sensitivity analyses we considered the potential for the presence of a 1 or 2 week lag between change in search volumes and change in rates of ED visits for mental health or suicide attempt. Specifically, we used a generalized linear model with a log link function to quantify the relative change in ED visits associated with searches the same week, the previous week, and 2 weeks earlier. We fit separate models for each search concept. All analyses were conducted using R (version: 4.0.2). The code to replicate these analyses is publicly available via GitHub at https://github.com/anthonysun95/Google_SSD_and_Mental_Health.

## Results

### Searches related to anxiety, depression, and suicidal ideation

**Figure 1:**
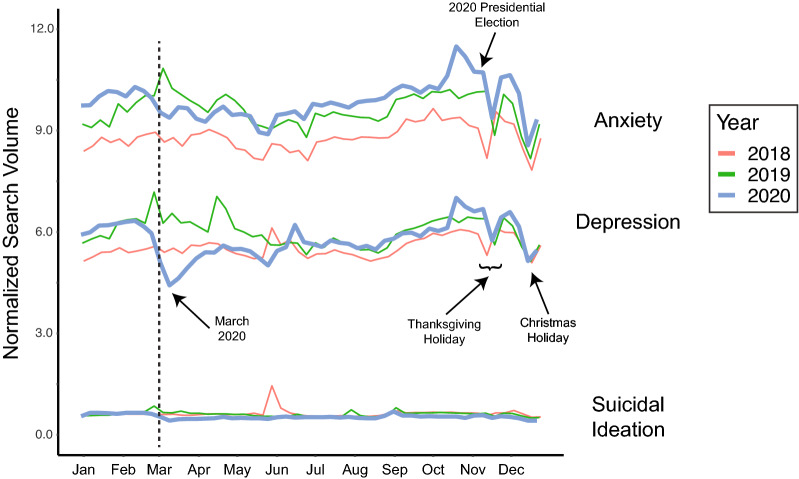
Yearly trends in normalized search volumes as indicated by Google’s COVID-19 Search Trends symptoms dataset (SSD) from 2018 to 2020 for anxiety, depression, and suicidal Ideation. Vertical dashed line denotes March 1. Noteworthy trends in search patterns include seasonal variation in searches for anxiety and depression, a decrease in searches for anxiety and depression coincident with the US Thanksgiving and Christmas holidays, an uptick in searches related to suicidal ideation coincident with the suicides of two celebrity figures in June 2018. *SSD* Google Symptom Search Dataset.

Internet searches related to anxiety were more common than searches related to depression, while searches related to suicidal ideation were less common (Fig. 1). Noteworthy trends include seasonal variation in searches related to anxiety and depression, a decrease in searches for anxiety and depression coincident with the US Thanksgiving and Christmas holidays, an uptick in searches related to suicidal ideation coincident with the suicides of two celebrity figures in June 2018 (Kate Spade and Anthony Bourdain), and an increase in searches for anxiety and depression coincident with the US presidential election in late October/early November 2020. For example, the Thanksgiving holiday was associated with a seasonally-adjusted 8.0% (95% CI: 3.4%, 12.6%) and 7.2% (95% CI: − 0.1%, 14.3%) decrease in searches related to anxiety and depression, respectively, and the Christmas holiday was associated with a seasonally-adjusted 14.5% (95% CI: 10.0%, 19.1%) and 14.6% (95% CI: 7.3%, 21.7%) decrease in searches related to anxiety and depression, respectively.

### COVID-19 pandemic and markers of mental health: associations within and between data sources

**Figure 2:**
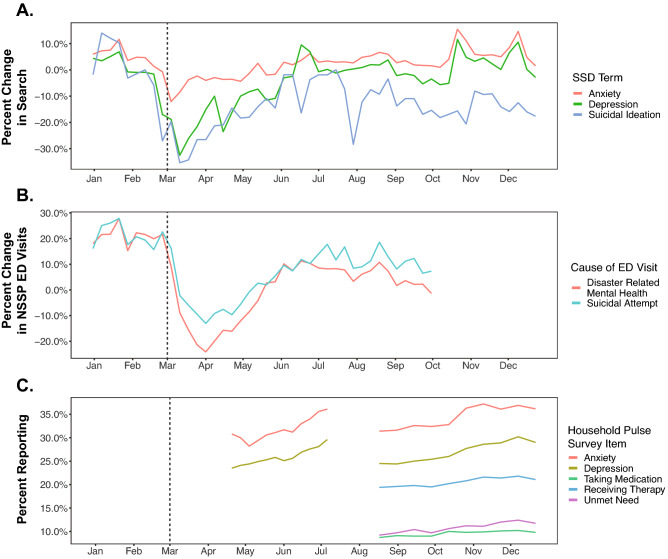
Impact of the COVID-19 Pandemic on markers of population mental health. (**A**) Relative change in normalized volume of Google searches for anxiety, depression, and suicidal ideation. (**B**) Relative change in ED visits for disaster-related mental health symptoms and suicide attempts from the NSSP dataset. Relative change in panels (**A**) and (**B**) is calculated as the change in 2020 relative to the corresponding weekly value in 2019, and expressed as a percentage of the 2019 value. (**C**) Self reported symptoms of anxiety and depression and mental health care or needs from the US Census HPS. Vertical dashed line denotes March 1, 2020. *SSD* Google Symptom Search Dataset, *ED* emergency department, *NSSP* National Syndromic Surveillance Program, *HPS* Household Pulse Survey.

In March 2020, the relative volume of searches related to anxiety, depression, and suicidal ideation decreased sharply, coincident with the enactment of pandemic-related restrictions across much of the country. Specifically, relative to the same time period in 2019, searches related to depression, anxiety, and suicidal ideation exhibited a pronounced *decrease*. Searches related to suicidal ideation showed a larger relative decrease (~ 30% decrease) than searches for anxiety (~ 10% decrease) (Fig. 2A). In the first 4 weeks of March 2020, searches related to anxiety, depression, and suicidal ideation decreased by a seasonally-adjusted average of 3.8% (95% CI: − 0.2%, 7.7%), 16.1% (95% CI: 9.6%, 22.4%) and 8.0% (95% CI: − 9.5%, 24.8%), respectively. Search volumes for these topics returned to pre-pandemic levels by the end of 2020.

A similar pattern was evident in the year-on-year change in rates of ED visits for mental health symptoms and suicide attempts in the NSSP data (Fig. 2B). Specifically, in the immediate aftermath of stay-at-home orders in March 2020, ED visits for mental health symptoms and suicidal attempts, and Internet searches for anxiety, depression, and suicidal ideation decreased sharply through mid-May and then generally increased towards pre-pandemic levels through the end of 2020. The HPS mirrors these results as well by showing increasing self-reported anxiety and depression starting around May 2020 (Fig. 2C).

**Figure 3:**
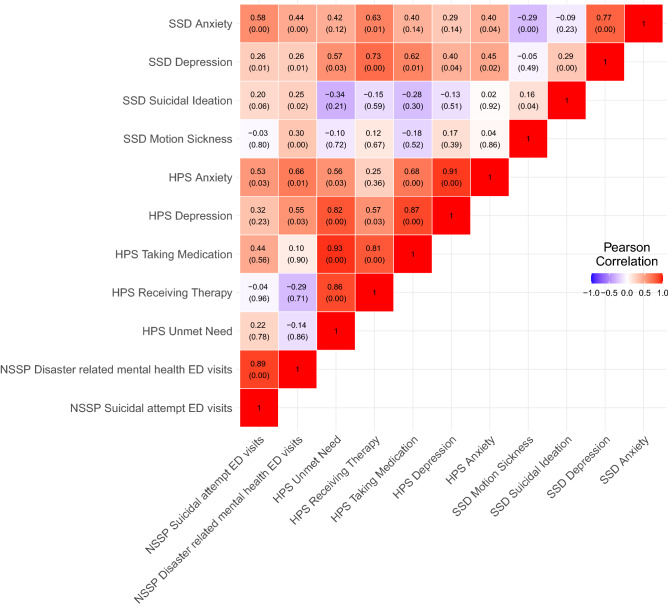
Pairwise Pearson correlation coefficients between normalized search volume for anxiety, depression, and suicidal ideation from the Google SSD, ED visits for mental health symptoms and suicide attempts from the NSSP, and self-reported anxiety, depression, and mental health treatment from the US Census Household Pulse Survey. *p* values for each correlation coefficient is presented in parentheses below it. Note that the NSSP and Household Pulse Survey datasets used in this study overlapped by only 7 weeks and these pairs of correlations should be interpreted with caution. *SSD* Google Symptom Search Dataset, *ED* emergency department, *NSSP* National Syndromic Surveillance Program, *HPS* Household Pulse Survey.

We used pair-wise correlation coefficients to quantify the similarity of these markers within and across data sources (Fig. 3). Measures within each data source tended to correlate very highly with each other. For example, searches related to depression and anxiety exhibited a Pearson correlation coefficient of 0.77 (*p* < 0.01). Rates of ED visits for mental health and suicide attempts were also strongly correlated (r = 0.89, *p* < 0.01). However, Internet searches related to motion sickness (used as a putative negative control unrelated to mental health) showed weak or negative correlations with Internet searches related to anxiety and depression.

**Figure 4:**
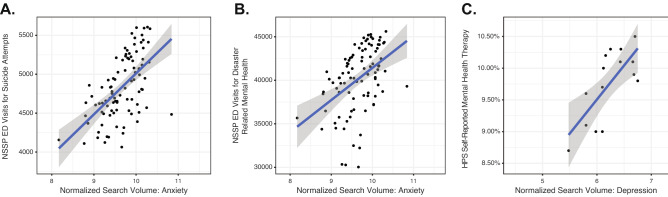
Scatterplots and superimposed best-fit lines showing select associations between (A) NSSP ED visits for suicide attempts and normalized SSD search volumes related to anxiety, (B) NSSP ED visits for disaster related mental health issues and normalized SSD search volumes related to anxiety, and (C) HPS self-reported mental health therapy use and normalized SSD search volumes related to depression. *SSD* Google Symptom Search Dataset, *ED* emergency department, *NSSP* National Syndromic Surveillance Program, *HPS* Household Pulse Survey.

Across data sources, Internet searches for anxiety were correlated with ED visits for suicide attempts (r = 0.58, *p* < 0.01), ED visits for mental health symptoms (r = 0.44, *p* < 0.01), and self-reported anxiety and mental health treatment (correlation coefficients ranging from 0.40 to 0.63). Internet searches for depression were most strongly correlated with the proportion of individuals responding to the HPS that self-reported use of mental health therapy (r = 0.73, *p* < 0.01), self-reported medication (r = 0.62, *p* = 0.01), and self-reported unmet need for mental health treatment (r = 0.57, *p* = 0.03). Bivariable scatter plots suggest that these relationships are approximately linear (Fig. 4). Searches related to suicidal ideation or motion sickness tended to be weakly associated with ED visits or self-reported survey measures. As the NSSP and HPS datasets used in this study overlapped by only about 7 weeks, we chose not to interpret these correlations.

In sensitivity analysis, we fit separate generalized linear models to quantify the (potentially lagged) association between searches related to anxiety, depression, and suicidal ideation on ED visits related to mental health and suicide attempts (Table 2). Searches related to anxiety were most strongly associated with ED visits for mental health and suicide attempts the same week. In contrast, searches related to depression exhibited similar associations with ED visits for mental and suicide attempts in the same and subsequent weeks.

**Table 2:** Percent change in the rate of emergency department (ED) visits for mental health and suicide attempts associated with 1 unit increase in the normalized volume of Internet searches related to anxiety, depression, or suicidal ideation.

ED visits	Searches	Searches in the same week	Searches 1 week prior the ED visits	Searches 2 weeks prior the ED visits
ED visits related to mental health	Anxiety	8.4% (2.7%, 14.4%)	1.8% (− 4.2%, 8.2%)	− 0.2% (− 5.0%, 4.9%)
	Depression	2.0% (− 3.9%, 8.1%)	3.4% (− 3.7%, 10.9%)	0.8% (− 4.9%, 6.8%)
	Suicidal ideation	21.8% (− 12.4%, 67.9%)	18.1% (− 19.8%, 71.5%)	− 3.7% (− 30.9%, 33.4%)
ED visits related to suicide attempt	Anxiety	9.7% (5.0%, 14.7%)	1.9% (− 3.1%, 7.2%)	0.4% (− 3.6%, 4.6%)
	Depression	2.9% (− 2.5%, 8.6%)	2.4% (− 4.0%, 9.1%)	− 0.2% (− 5.4%, 5.2%)
	Suicidal ideation	21.7% (− 9.9%, 63.4%)	8.9% (− 23.6%, 53.7%)	− 8.1% (− 32.3%, 24.2%)

Results are from a generalized linear model with a log link function and simultaneously estimating the association with normalized search volume the same week, 1 week prior, and 2 weeks prior.

## Discussion

There are currently no systems that allow for continuous assessment of population mental health across the US in near real-time. Aiming to address this shortcoming, we compared Internet searches for anxiety, depression, and suicidal ideation to markers of mental health from the CDC’s NSSP and the US Census HPS. We found that Internet searches related to anxiety correlated strongly with ED visits for mental health symptoms and suicide attempts (Figs. 2, 3). We also found that Internet searches related to depression, and to a lesser degree, searches for anxiety, were correlated with self-reported need for or receipt of treatment (prescription medication/counseling or therapy) in a population-based sample. Internet searches related to suicidal ideation did not correlate with searches for anxiety or depression, with ED visits, or with self-reported symptoms.

With respect to the COVID-19 pandemic, the observed Internet search patterns could be interpreted as suggesting that mental illness writ large and across the broad population did not increase dramatically during the first phase of the pandemic in the US and may have decreased somewhat in early March compared to pre-pandemic levels. However, as the pandemic and associated public health restrictions persisted, population mental health may have worsened over the summer and fall of 2020, returning essentially to pre-pandemic levels. This interpretation of the results and a fine-grained understanding of the longitudinal impact of the pandemic using weekly search data provides nuance to the mix of findings that suggest worsening mental health in some cases^13–16^, but not in others^19–22,36^. Moreover, some studies have indicated that specific subpopulations such as adolescent females or Hispanic populations^37,38^ may have had increases in mental health challenges during this time period, even if the population at large did not evince such trends. Such findings imply that long-standing health disparities may have been exacerbated in particular by the pandemic.

More broadly, our results raise the possibility that aggregated and anonymized Internet search queries can be used as a near real time and easily accessible indicator of the state of population mental health. A large number of people use Google’s search engine, with an important fraction of searches indicative of people’s interest in or concern for their physical or mental health. Internet search patterns have been previously used to identify food establishments likely to have food safety violations^39^, to assess spatial patterns of the prevalence of Lyme disease^40^, and to forecast COVID-19 cases and deaths^41^, among other examples^42,43^.

However, there have been few attempts to use aggregate search queries to gain novel insights into population mental health and results of previous efforts have been mixed^25,26,44,45^. Google is currently publishing SSD data for the US and selected other countries with an average latency of less than a week and at county-level (or equivalent) geographic granularity, including data from well before the current pandemic. Unlike formal surveys that require the deployment of standardized questionnaires or interviews, dedicated workforces, and complex logistics (which can be expensive and difficult to undertake), data on Internet search patterns may provide a rapid and easily accessible alternative to gauge population-level interests or concerns in numerous locations simultaneously. If Internet search activity provides a marker of population health, at least under certain conditions, then rapidly available data on aggregated search patterns could serve to complement existing public health syndromic surveillance systems.

We also observed interesting associations between other events of national importance and Internet searches related to depression and anxiety. For example, in the time period immediately prior to the 2020 US presidential elections, search queries related to anxiety and depression showed increases, similarly to self-reported use of prescription medication for mental health, or therapy, and counseling. Analogously, both Internet searches for depression and anxiety and ED visits related to anxiety, depression, and suicidal ideation decreased notably during the weeks of Thanksgiving and Christmas.

Our study has several important limitations. First, we used data from users of Google’s search engine, which represents a subset of the entire population. Not all individuals with symptoms or concerns will perform an Internet search, either due to personal choice or lack of reliable Internet access. Thus, at best, search patterns may reflect the concerns and interests of a selected subset of individuals in any given place and time, especially those with higher income levels^46^. The same caveat regarding representativeness of the results applies to most surveys, the results of which must be interpreted within the context of the survey’s sampling frame and potential for distortion from non-response. Second, search patterns may also be influenced by external events and news reports. For example, the volume of searches for anosmia (i.e., the loss of the sense of smell) increased sharply following media reports suggesting anosmia as a symptom of COVID-19 starting on March 23, 2020^30,47^. The development of methods that remove the influence of news and other external events from search patterns is an active area of research^41^. Third, only searches in English and Spanish are captured by SSD, potentially excluding relevant searches in other languages. Fourth, the application of differential privacy inevitably adds some stochastic noise to the search data and limits the number of queries any one user may contribute. However, this latter feature is also a strength of the dataset as it limits the influence on the data of healthcare professionals or others that may search more frequently for other purposes. Finally, we chose a priori to focus on searches related to anxiety, depression, and suicidal ideation. Although beyond the scope of this project, a more comprehensive approach considering search volumes related to any or all of the 420 conditions/symptoms available (and potentially other external data) and using a machine-learned prediction model may have resulted in better prediction of population mental health.

Given the national scale of the datasets we used in this study and the robust correlations documented among them, these results suggest that Internet search may serve as a proxy for important and relevant behaviors, feelings, or concerns in the population. Our results also suggest that the Internet may be an important resource for individuals with mental health concerns, especially in times of crises such as the coronavirus pandemic. Specifically, Internet searches appear to correlate strongly with a variety of help-seeking behaviors—ranging from self-reported use of therapy or medication to mental-health ED visits—suggesting that search may be capturing a spectrum of population mental health needs, rather than only capturing severe (or conversely, minor) mental health problems. Indeed, recent trends in psychiatry and psychology have emphasized dimensional models of mental health (e.g., Research Domain Criteria (RDoC)^48^), which underscore that mental health problems are continuous in nature rather than being restricted to binary categories. These data therefore suggest that SSD may be a useful marker of mental health concerns or needs in populations, particularly when used in concert with other data sources such as NSSP and HPS.

## Data Availability

The anonymized and aggregated search data analyzed herein was obtained from the publicly-available Google COVID-19 Symptom Search Trends (published at https://pair-code.github.io/covid19_symptom_dataset/?country=US). The data analyzed in this paper consisted of anonymized, aggregated, and differentially private counts of searches for different symptoms and conditions. Data from the NSSP were those previously published by Holland et al. (2021)^20^ and reused in the current study with permission from the authors. Data from the Household Pulse Survey are publicly available (published at https://www.cdc.gov/nchs/covid19/pulse/mental-health.htm).
